# Hypoxia-Induced Mitogenic Factor (HIMF/FIZZ1/RELMα) Recruits Bone Marrow-Derived Cells to the Murine Pulmonary Vasculature

**DOI:** 10.1371/journal.pone.0011251

**Published:** 2010-06-22

**Authors:** Daniel J. Angelini, Qingning Su, Irina A. Kolosova, Chunling Fan, John T. Skinner, Kazuyo Yamaji-Kegan, Michael Collector, Saul J. Sharkis, Roger A. Johns

**Affiliations:** 1 Department of Anesthesiology and Critical Care Medicine, Johns Hopkins University School of Medicine, Baltimore, Maryland, United States of America; 2 Department of Oncology and Cancer Biology, Johns Hopkins University School of Medicine, Baltimore, Maryland, United States of America; 3 Division of Pulmonary and Critical Care Medicine, Department of Medicine, Johns Hopkins University School of Medicine, Baltimore, Maryland, United States of America; University of Illinois at Chicago, United States of America

## Abstract

**Background:**

Pulmonary hypertension (PH) is a disease of multiple etiologies with several common pathological features, including inflammation and pulmonary vascular remodeling. Recent evidence has suggested a potential role for the recruitment of bone marrow-derived (BMD) progenitor cells to this remodeling process. We recently demonstrated that hypoxia-induced mitogenic factor (HIMF/FIZZ1/RELMα) is chemotactic to murine bone marrow cells *in vitro* and involved in pulmonary vascular remodeling *in vivo*.

**Methodology/Principal Findings:**

We used a mouse bone marrow transplant model in which lethally irradiated mice were rescued with bone marrow transplanted from green fluorescent protein (GFP)^+^ transgenic mice to determine the role of HIMF in recruiting BMD cells to the lung vasculature during PH development. Exposure to chronic hypoxia and pulmonary gene transfer of HIMF were used to induce PH. Both models resulted in markedly increased numbers of BMD cells in and around the pulmonary vasculature; in several neomuscularized small (∼20 µm) capillary-like vessels, an entirely new medial wall was made up of these cells. We found these GFP^+^ BMD cells to be positive for stem cell antigen-1 and c-kit, but negative for CD31 and CD34. Several of the GFP^+^ cells that localized to the pulmonary vasculature were α-smooth muscle actin^+^ and localized to the media layer of the vessels. This finding suggests that these cells are of mesenchymal origin and differentiate toward myofibroblast and vascular smooth muscle. Structural location in the media of small vessels suggests a functional role in the lung vasculature. To examine a potential mechanism for HIMF-dependent recruitment of mesenchymal stem cells to the pulmonary vasculature, we performed a cell migration assay using cultured human mesenchymal stem cells (HMSCs). The addition of recombinant HIMF induced migration of HMSCs in a phosphoinosotide-3-kinase-dependent manner.

**Conclusions/Significance:**

These results demonstrate HIMF-dependent recruitment of BMD mesenchymal-like cells to the remodeling pulmonary vasculature.

## Introduction

Hypoxia-induced mitogenic factor (HIMF), also known as “found in inflammatory zone 1” (FIZZ1) and resistin-like molecule alpha (RELMα), is a pleiotropic cytokine that is highly inducible in lung [1]–[3]. We have shown it to have mitogenic, angiogenic, vasoconstrictive, and chemokine-like properties [3]–[6]. We initially described HIMF in the remodeling vasculature of the chronic hypoxia model of pulmonary hypertension (PH) [3] and have recently determined that HIMF plays a critical role in this process [7]. In rats, *in vivo* knockdown of HIMF specifically in the lung reduces the mean pulmonary artery pressure, pulmonary vascular resistance, and vascular remodeling associated with chronic hypoxia, whereas pulmonary gene transfer of HIMF initiates vascular remodeling and increases these physiological measurements [7]. Liu et al. [8] has shown that HIMF plays a key role in the transition of fibroblasts to myofibroblasts, which is essential to bleomycin-induced fibrosis and may play a role in vascular remodeling associated with PH. Our lab and others have demonstrated that the addition of recombinant HIMF to cultured cells activates the phosphoinosotide-3-kinase (PI-3K)/Akt and extracellular signal-regulated kinase 1/2 (ERK1/2) mitogen-activated kinase (p42/44 MAPK) pathways in several different cell types [3], [9], [10]. Finally, we have demonstrated that HIMF is chemotactic for undifferentiated murine bone marrow-derived (BMD) cells and this action is mediated through Bruton's tyrosine kinase (BTK) [5].

Pulmonary vascular remodeling is a key component of the pathogenesis of PH. Recent evidence has suggested the possibility that BMD progenitor cells are recruited during this remodeling process [11], [12]. Davie *et al.* [11] demonstrated that BMD c-kit^+^ cells were localized within the pulmonary artery walls of chronically hypoxic calves, and Spees *et al.* [12] reported that α-smooth muscle actin (α-SMA)^+^ BMD cells became engrafted into the pulmonary vasculature in an inflammatory model of PH. These studies suggest the interesting possibility that pulmonary vascular remodeling may involve cells of multiple origins, possibly including multipotent “stem cells.”

In the current study, we demonstrate in mice that both chronic hypoxia and pulmonary gene transfer of HIMF induce BMD cell recruitment to the remodeling pulmonary vasculature; many of these cells localize to the newly formed media of previously non-muscularized capillary-like vessels. Both mouse models led to significant pulmonary vascular remodeling consistent with our prior demonstration of structural and hemodynamic PH. We describe several of these cells to be stem cell antigen (sca)-1^+^ and c-kit^+^ as well as CD31^−^ and CD34^−^. The BMD cells located within the vessel walls are likely of mesenchymal origin as they are α-SMA^+^. We also show that HIMF induces migration of human mesenchymal stem cells (HMSCs) in a PI-3K-dependent manner *in vitro*. All of these data suggest that HIMF/FIZZ1/RELMα recruits BMD cells to the remodeling pulmonary vasculature.

## Materials and Methods

### Experimental animals/bone marrow transplantation

Female C57/BL6 mice (6–8 weeks old; Charles River Laboratories, Wilmington, MA) were used as bone marrow transplant recipients for all experiments. Four- to 6-week-old male transgenic enhanced green fluorescent protein (EGFP) mice on a C57BL/6 background (Jackson Laboratories, Bar Harbor, ME; stock number: 003291) were used as bone marrow donors [13]. Animal housing and experimental protocols were approved by the Animal Care and Use Committee of the Johns Hopkins University (Protocol #: MO08M424). The mice were given free access to food and water and were maintained in a room with a 12∶12 hour light-dark cycle between 20–24°C. Bone marrow transplants were performed as previously stated [14]. Briefly, whole bone marrow was collected from the transgenic EGFP mice, and 2×10^6^ bone marrow cells were transplanted into lethally irradiated (1,050 cGy) recipient mice through intravenous injection. The transplant recipients were then allowed to recover for 4–6 weeks.

### Antibodies and Inhibitors

Rabbit anti-mouse HIMF polyclonal antibodies were prepared as we have described [3]. Polyclonal rabbit anti-GFP antibodies were purchased from Invitrogen (Carlsbad, CA). Goat anti-mouse RELMα (HIMF), rat anti-mouse c-kit, and rat anti-mouse sca-1 antibodies were purchased from R&D Systems (Minneapolis, MN). The rat anti-mouse CD31 and the mouse anti-β-actin monoclonal antibodies were purchased from BD Biosciences (San Jose, CA). The rabbit anti-CD34 antibody was purchased from Santa Cruz Biotechnology (Santa Cruz, CA). The mouse anti-α-SMA antibody was purchased from DakoCytomation (Carpinteria, CA). Fluorescein isothiocyanate (FITC), rhodamine, cy3- and cy5-labeled secondary antibodies were purchased from Jackson ImmunoResearch (West Grove, PA). The rabbit anti-phospho-Akt (Ser473/Thr308) and rabbit anti-phospho-ERK1/2 (Thr202/Tyr204) polyclonal antibodies as well as the pharmacological inhibitors U0126 and LY294002 were purchased from Cell Signaling Technologies, Inc. (Beverly, MA).

### Intranasal instillation of viral vectors

To selectively induce HIMF expression in the lungs, we used a recombinant adeno-associated virus (AAV) vector that expresses murine HIMF (AAV-HIMF) as stated [7]. This viral vector contains the ubiquitous CB promoter and was prepared by the University of Florida Vector Core Laboratory. To control for the possibility of viral effects, we used a similar empty AAV vector (AAV-null). Intranasal instillation of the AAV vectors was performed as follows. First, female bone marrow transplant recipient mice were lightly anesthetized with isoflurane. Then, a gel loading tip primed with 50 µL of solution that contained either 2.5×10^10^ viral particles (VP) AAV-HIMF [with 5 µL lipofectamine 2000 (Invitrogen)] or 2.5×10^10^ VP AAV-null (with 5 µL lipofectamine 2000) was placed directly on the nasal passage and the solution expelled. Mice were sacrificed 14 days after intranasal instillation of the vector by isoflurane overdose, and tissue was processed as stated [7]. Briefly, the heart and lungs were removed *en bloc*. The right lung was tied off and the left lung was inflated with 1% low-melting point agarose with constant pressure and then placed on ice. The right lung was removed and split into individual lobes. A portion of the right lung was frozen in liquid N_2_ and stored at −80°C for use in Western blot analysis. The agarose-filled lung tissue was fixed in 4% paraformaldehyde and then processed for either paraffin or frozen sections as stated [3], [7]. The efficiency of instillation was measured by Western blotting or immunohistochemistry for HIMF.

### Chronic hypoxia model of PH

Female mice receiving bone marrow transplantation were exposed to either normal room air (20.8% O_2_) or 10% O_2_ for 7 days as we have described [3], [7], [15]–[18]. The fractional concentration of O_2_ was monitored and controlled with a Pro∶Ox model 350 unit (Biospherix, Redfield, NY) by infusion of N_2_ (Roberts Oxygen, Rockville, MD) balanced against an inward leak of air through holes in the chamber. The chambers were continuously scavenged for CO_2_ and ammonia. At the end of the 7-day period, mice were sacrificed and processed as stated above.

### Immunohistochemistry

Paraffin blocks of lungs from mice exposed to room air (20.8%O_2_), hypoxia (10.0% O_2_), AAV-null, or AAV-HIMF were cut into 6-µm sections and placed onto clean glass slides. The slides were then deparaffinized and rehydrated as described previously [3], [7]. For antigen retrieval, the slides were submerged in antigen unmasking solution (Vector Laboratories, Burlingame, CA) and heated at 95°C for 20 min. Endogenous peroxidase activity was blocked by treatment with 3% H_2_O_2_ in PBS for 10 min at room temperature (RT). We then blocked endogenous avidin and biotin for 15 min each at RT using the Avidin/Biotin Blocking Kit (Vector Laboratories). Nonspecific protein binding was blocked by treatment with either normal goat or horse serum for 30 min at RT. After the blocking steps, the sections were treated with polyclonal rabbit anti-GFP antibodies, polyclonal goat anti-HIMF antibodies, or antibody diluent alone for 2 h at RT. The slides were then washed with PBS and treated with either goat anti-rabbit or donkey anti-goat biotinylated secondary antibodies (Vector Laboratories) in PBS for 30 min at RT. Then, the lung sections were exposed to an ABC horseradish peroxidase (HRP) reagent (Vector Laboratories) for 30 min at RT. The GFP/HIMF signal was developed with the Peroxidase Substrate Kit DAB (Vector Laboratories). The sections were counterstained with hematoxylin as we have described [7]. Finally, the sections were dehydrated, cleared, and mounted with Cytoseal 60 (Richard-Allan Scientific, Kalamazoo, MI). The stained sections were visualized with an Olympus-BHS microscope attached to a QImaging Retiga 4000RV digital camera. Images were captured with ImagePro Plus (version 5.1) software.

### Quantification of GFP-positive cells

To quantify the number of GFP^+^ cells associated with the pulmonary vasculature, the GFP-stained lung sections were examined with a 40× objective lens. Peripheral pulmonary arteries associated with alveolar sacs and alveolar ducts with an external diameter between 25 and 100 µm were identified, and the associated GFP^+^ cells were counted. GFP^+^ cells were quantified in approximately 50 consecutive vessels per animal. The values are expressed as the mean number of GFP^+^ cells per vessel (mean ± SEM).

### Assessment of pulmonary vascular remodeling

Pulmonary vascular remodeling of the mice was analyzed as we have previously described [7], [15]–[18]. The murine lung sections were initially evaluated after being stained with hematoxylin and eosin. Lung sections were also dual stained with von Willebrand Factor (endothelium) and α-SMA (vascular smooth muscle) as we have described [7], [17], [18]. Upon completion of the dual-stain, approximately 100 randomly selected arteries were examined under an Olympus-BHS microscope attached to a QImaging Retiga 4000RV digital camera. Only arteries with an internal diameter of <80 µm were examined. These vessels were classified as non-muscular (NM), partially-muscular (PM), or fully-muscular (FM), according to α-SMA staining. Vessels that had at least one α-SMA^+^ cell but lacked a continuous layer were considered PM. FM vessels had a continuous α-SMA band. These vessels were then analyzed as we have described [7], [17], [18]. Images of dual-stained sections were captured as stated above.

### Immunofluorescence microscopy

Frozen lung sections were air dried for 30 min, permeabilized with 0.2% Triton X-100/PBS, and then blocked with 2% BSA/PBS. The sections were exposed to primary antibody (c-kit, sca-1, CD31, CD34, α-SMA, or HIMF) followed by the appropriate secondary antibody (cy3-donkey anti-rabbit IgG, cy3-donkey anti-rat IgG, FITC-donkey anti-mouse IgG, or cy5-donkey anti-mouse IgG). Nuclei were stained with 50 ng/mL 4′,6′-diamidino-2-phenylindole dilactate (DAPI) for 5 min. Staining was imaged with a Zeiss 510 Meta confocal microscope via a 20× lens. When four channels were used, cy5 was labeled with pink. Differential interference contrast imaging was used to show the tissue structure.

### *In vitro* Cell Migration Assay

HMSCs were purchased from Lonza (Walkersville, MD) and cultured according to the manufacturer's specifications. Only HMSCs from *passages* 3–5 were used. Costar 24-well cell migration plates with polycarbonate membranes with 8-µm pore size (Costar Corporation, Cambridge, MA) were used for this assay. The lower chamber was filled with 0.6 mL of medium with or without 100 nM recombinant HIMF. Then, 100 µL of HMSC suspension (10^5^ cells) was added to the upper chamber. In some experiments, the cells were pretreated for 30 min with vehicle (0.1% DMSO) or a pharmacological kinase inhibitor [U0126 (10 µM) or LY294002 (10 µM)]. After 24 h at 37°C, the cells were removed from the top surface of the membrane. Migrated cells on the bottom surface were stained with Coomassie blue. The average number of cells per field was evaluated under an Olympus-BHS microscope. Images were captured with a QImaging Retiga 4000RV digital camera, analyzed by NIH ImageJ software, and reported as the number of positively stained pixels versus the total number of image pixels.

### Western Blot Analysis

HMSCs were cultured to approximately 70% confluence and then serum- and growth factor- starved overnight. Then they were treated with vehicle or 100 nM HIMF for various time periods in the presence or absence U0126 (10 µM) or LY294002 (10 µM). The HMSCs were collected in equal volumes of Laemlli's sample buffer, resolved by 4–20% gradient sodium dodecyl sulfate-polyacrylamide gel electrophoresis (SDS-PAGE; Bio-Rad), and transferred to nitrocellulose membranes (Bio-Rad). The blots were blocked with 5% non-fat milk-TBS-T and incubated with either rabbit anti-phospho-Akt (Ser473/Thr308) or rabbit anti-phospho-ERK1/2 (Thr202/Tyr204) antibody. The blots were then incubated with anti-rabbit IgG conjugated to HRP antibodies, developed with enhanced chemiluminescence (ECL) and exposed to X-ray film (Denville Scientific; Metuchen, NJ). To ensure equal protein loading and transfer, the blots were stripped using the Blot Restore kit according to the manufacturer's instructions (Millipore; Billerica, MA), reprobed with mouse anti-β-actin antibodies and processed as stated above.

### Statistical analysis

A student's t-test was used to compare mean responses between individual experimental and control groups. ANOVA was used to compare the mean responses among experimental and control groups in experiments with multiple groups. The Dunnett and Scheffe F test was used to determine between which groups significant differences existed. A *P*-value <0.05 was considered significant for all experiments.

## Results

### Hypoxia and pulmonary HIMF gene transfer induce HIMF expression in bone marrow transplant recipient mice

**Figure 1:**
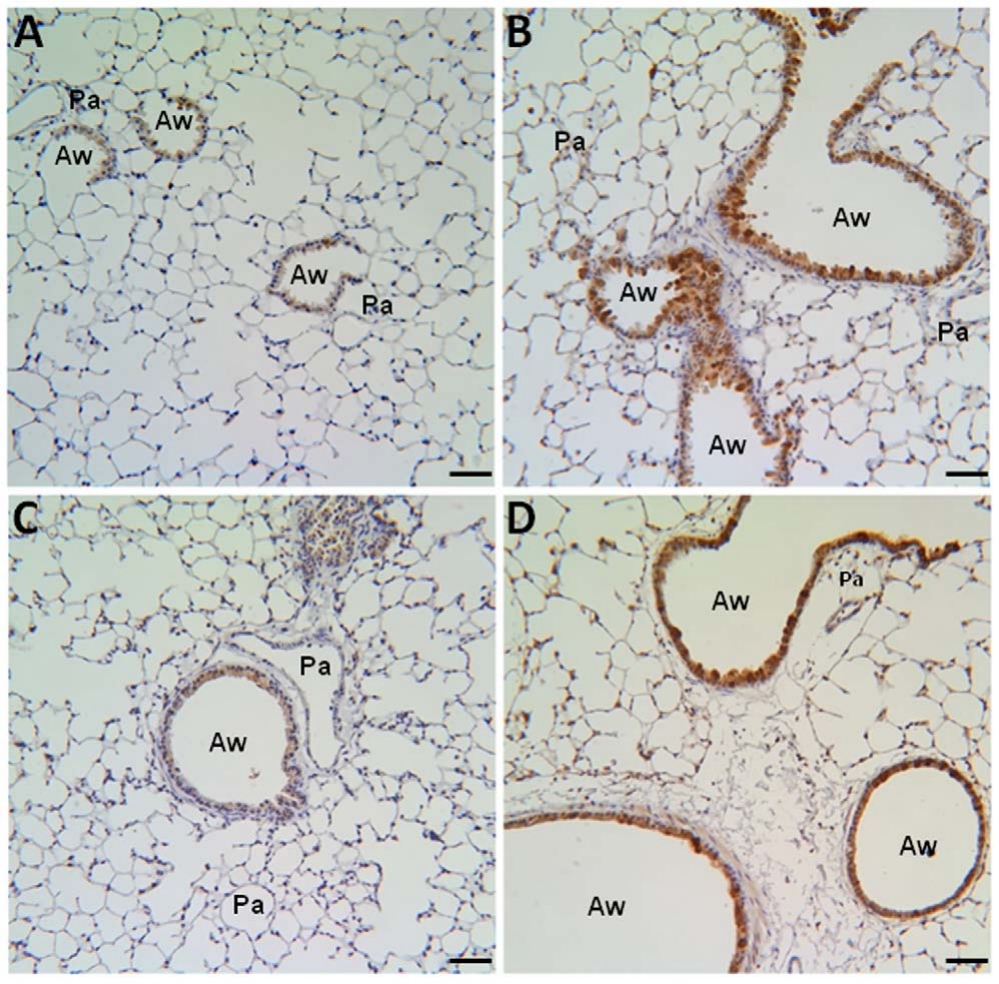
HIMF expression in murine lung. Paraffin-embedded lung sections from normoxic (7d, 20.8% O_2_) (A), hypoxic (7d, 10.0% O_2_) (B), AAV-null-treated (14d, 2.5×10^10^ VP) (C), and AAV-HIMF-treated (14d, 2.5×10^10^ VP) (D) mice were rehydrated and stained with goat anti-mouse HIMF polyclonal antibodies. Pa: pulmonary artery. Aw: airway. Scale bar: 50 µm.

To determine the expression pattern of HIMF in our bone marrow transplant recipients, we evaluated lung sections from mice exposed to normoxia (7d, 20.8% O_2_), hypoxia (7d, 10.0% O_2_), AAV-null (14d, 2.5×10^10^ VP), or AAV-HIMF (14d, 2.5×10^10^ VP) by immunohistochemistry. Both hypoxia and HIMF pulmonary gene transfer led to HIMF expression in the lungs of bone marrow transplant recipients (Figure 1). In normoxic lungs, HIMF staining was not apparent (Figure 1A), but HIMF was strongly expressed in the airway epithelium, pulmonary inflammatory cells, and pulmonary vasculature of hypoxic animals (Figure 1B). In the HIMF gene transfer experiments, HIMF staining was absent in AAV-null treated lungs (Figure 1C) but strong in airway epithelium and the pulmonary vasculature (Figure 1D). The introduction of AAV-null virus into the lungs did not change HIMF expression compared to normoxic control (Figure 1A, C).

### BMD cells are localized to the pulmonary vasculature

**Figure 2:**
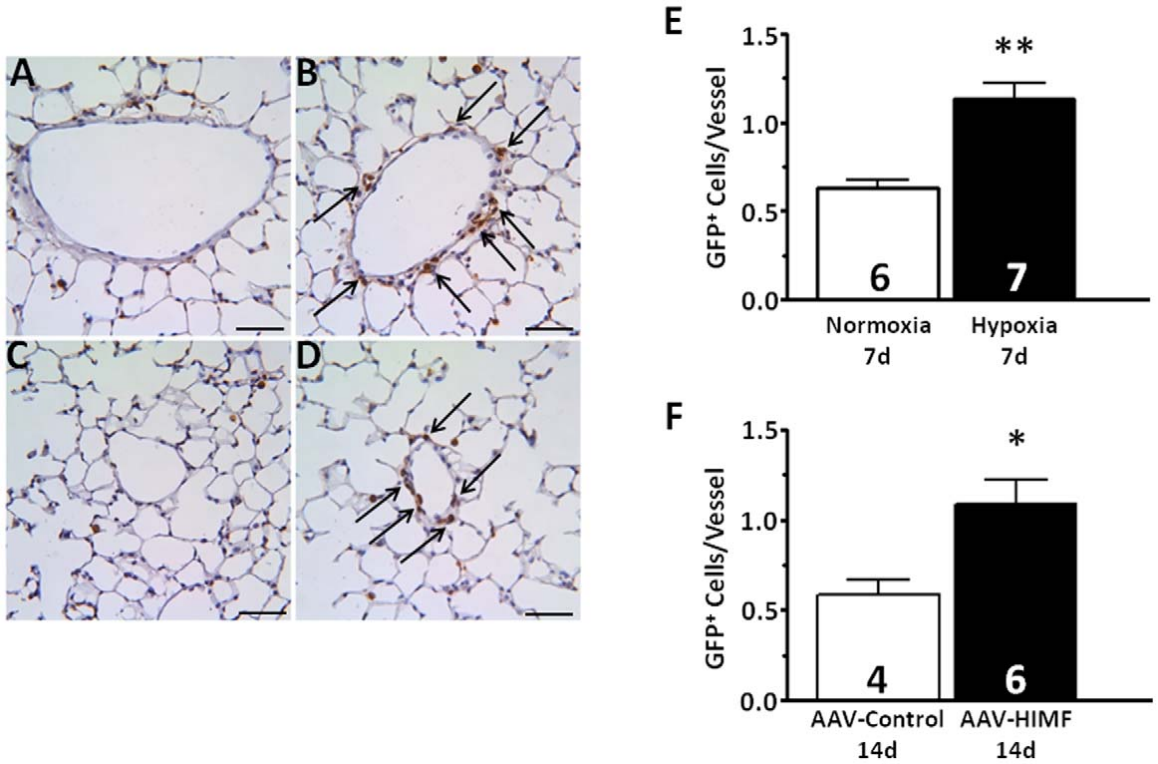
Chronic hypoxia and pulmonary HIMF gene transfer increase the number of BMD cells associated with the pulmonary vasculature. A–D: Paraffin-embedded lung sections from mice exposed to normoxia (7d, 20.8% O_2_) (A), hypoxia (7d, 10.0% O_2_) (B), AAV-null (14d, 2.5×10^10^ VP) (C), or AAV-HIMF (14d, 2.5×10^10^ VP) (D) were probed with polyclonal antibodies raised against GFP. Arrows indicate GFP^+^ cells within the vasculature. Scale bar: 50 µm. E, F: Quantification of GFP^+^ cells within the pulmonary vasculature. GFP^+^ cells within the pulmonary vasculature are shown as mean ± SEM of GFP^+^ cells/vessel. * *P*<0.05, ** *P*<0.01 vs. control.

**Figure 3:**
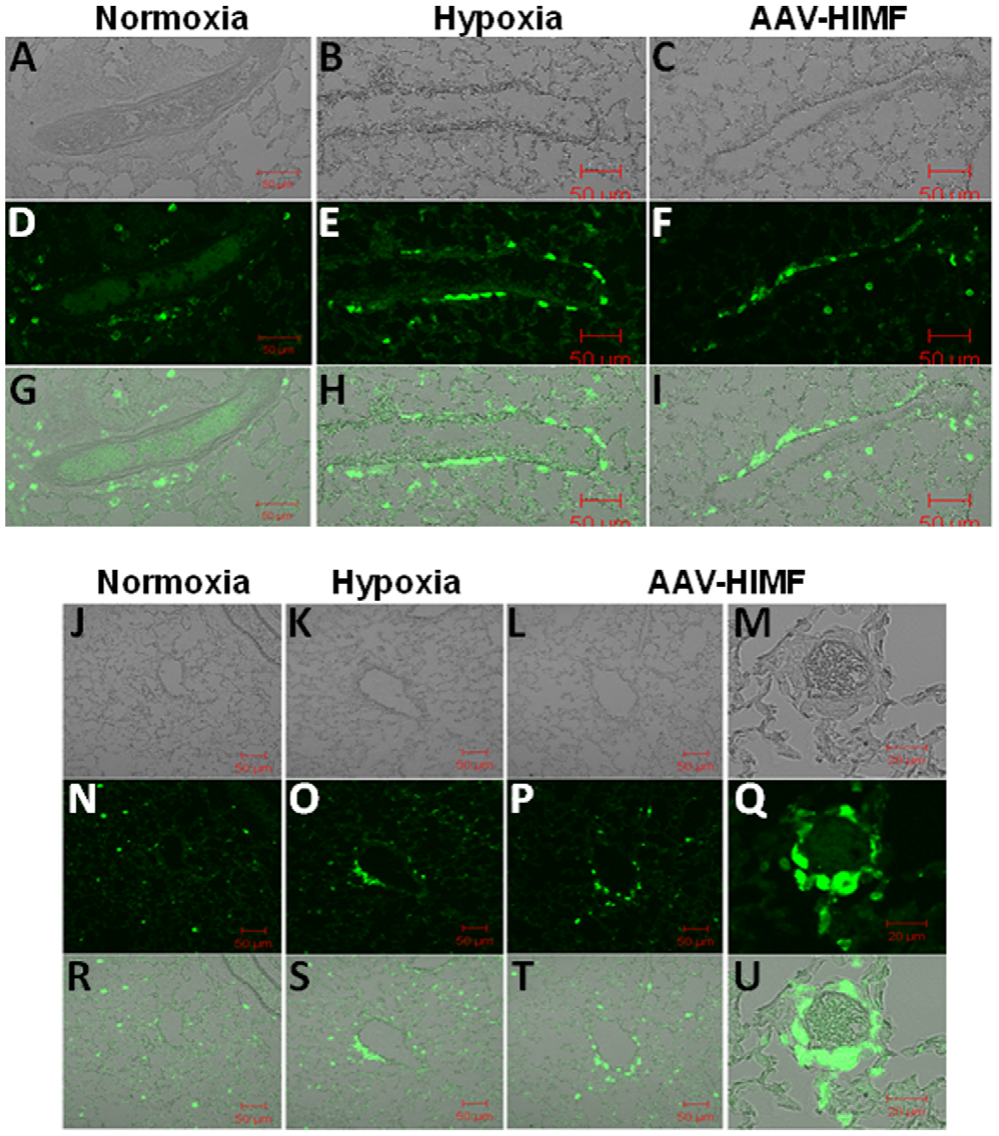
Both chronic hypoxia and pulmonary HIMF gene transfer recruit BMD cells to the pulmonary vasculature. (A–C, J–M) Light micrograph of fluorescence images to show blood vessel structure. Frozen sections from normoxic (20.8% O_2_) (D, N), hypoxic (10.0% O_2_) (E, O), and AAV-HIMF treated (2.5×10^10^ VP) (F, P, Q) lungs were stained with a rabbit anti-GFP polyclonal antibody that was visualized by an FITC-conjugated goat anti-rabbit IgG antibody (green). (G–I, R–U): Differential interference contrast images of light and fluorescence images to show structure. A–L, N–P, R–T: Scale bar: 50µm. M, Q, U: Scale bar: 20µm.

Immunostaining for GFP with subsequent quantitative analysis showed that in both hypoxic and AAV-HIMF-treated bone marrow transplant recipients, increased numbers of GFP^+^ cells became associated with the vasculature (Figure 2) compared to appropriate simultaneous controls. Visual examination of both hypoxic and AAV-HIMF-treated lung sections revealed GFP^+^ cells associated with the vasculature; in comparison, relatively few GFP^+^ cells were associated with the pulmonary vasculature of normoxic or AAV-null treated mice (Figure 2A–D). Notably, mice from all groups had some GFP^+^ cells evenly distributed throughout the lung parenchyma, most likely as a result of irradiation injury [19] or possibly representing normal distribution of inflammatory BMD cells in lung. Quantification of the GFP^+^ cells associated with individual pulmonary vessels confirmed the visual findings. Our analysis revealed approximately twice as many GFP^+^ cells/vessel in hypoxic mice (1.13±0.09 GFP^+^ cells/vessel) compared with normoxic mice (0.63±0.05 GFP^+^ cells/vessel; *P* = 0.0005; Figure 2E). Similarly, the number of GFP^+^ cells/vessel in AAV-HIMF-treated mice (1.09±0.14 GFP^+^ cells/vessel) was greater than that of AAV-null-treated mice (0.58±0.09 GFP^+^ cells/vessel; P = 0.0266; Figure 2F). The result of AAV-HIMF treatment looks strikingly similar with hypoxia; HIMF gene transfer was sufficient to induce the recruitment of BMD cells to the pulmonary vasculature (Figure 2). To gain a different visual perspective of these lung sections, we performed immunofluorescence microscopy using lung sections from each group. Figure 3A–I shows that after either hypoxia or AAV-HIMF treatment, GFP^+^ BMD cells formed an organized layer that surrounded the blood vessel. A cross sectional view is shown in Figure 3J–U. A higher magnification image of a small vessel from an AAV-HIMF treated mouse revealed that the entire media layer of the neomuscularized small vessel was made up of GFP^+^ BMD cells (Figure 3M, Q, U). Most of the recruitment of GFP^+^ cells was observed in small vessels approximately 20–80 µm in diameter, including small capillary sized vessels normally without any muscle cells. This is strong evidence of a functional role of these cells in the remodeling pulmonary vasculature and the development of PH.

### Hypoxia and AAV-HIMF treatment similarly increased pulmonary vascular remodeling

**Figure 4:**
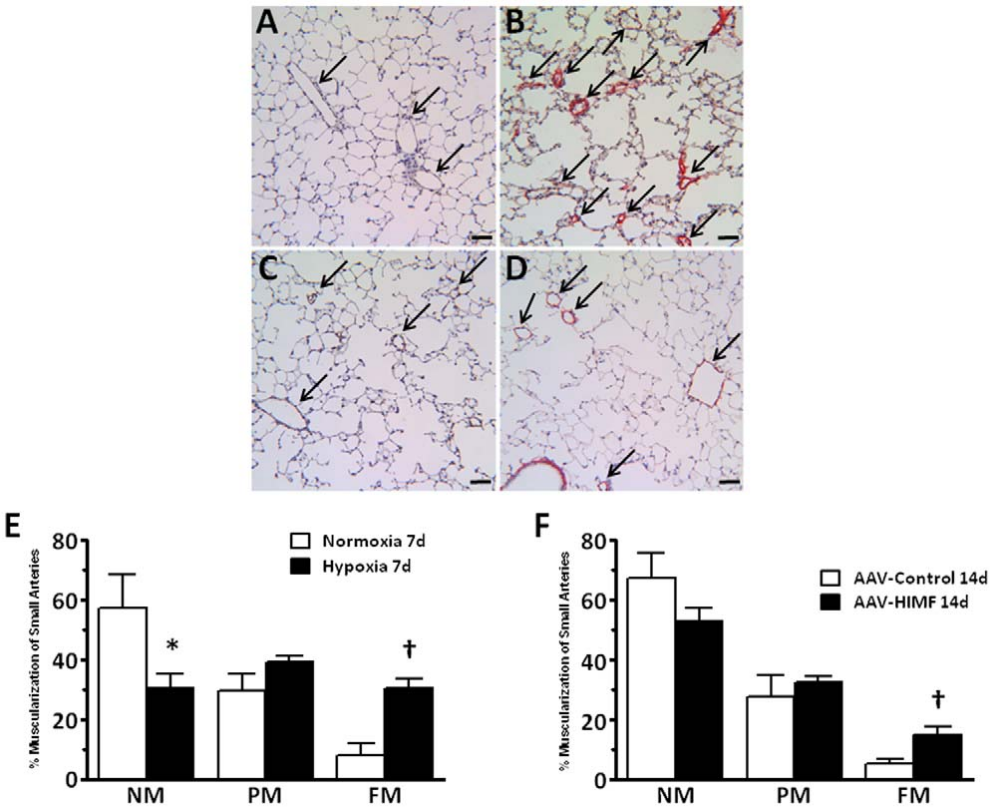
Both chronic hypoxia and pulmonary HIMF gene transfer induce pulmonary vascular remodeling. A–D: Paraffin-embedded lung sections were double-stained with antibodies to von Willebrand factor (black) and α-smooth muscle actin (red). Arrows indicate small pulmonary vessels. Scale bar: 50 µm. E, F: Percent muscularization of small pulmonary arteries in mouse lungs. NM, non-muscularized; PM, partially muscularized; FM, fully muscularized. *Significantly decreased vs. control at *P*<0.05. ^†^Significantly increased vs. control at *P*<0.05.

In the bone marrow transplant recipients, muscularization of the small pulmonary arteries was increased by both hypoxia and AAV-HIMF treatment compared to the corresponding controls (Figure 4A–D). After exposure to 10.0% O_2_ for 7 days, the percentage of vessels that were FM increased (30.31±3.46% vs. 7.96±4.30%; *P* = 0.0018), while the percentage of vessels that were NM decreased (30.52±4.78% vs. 57.46±11.19%; *P* = 0.0392) compared with control animals exposed to room air (Figure 4E). Examination of the lung sections 14 days after intranasal instillation of AAV-HIMF revealed increased percentages of FM vessels (15.05±11.19%) when compared to simultaneously treated AAV-null controls (5.29±2.64%; *P* = 0.0187; Figure 4F).

### BMD cells are recruited to the smooth muscle layer of pulmonary vessels

**Figure 5:**
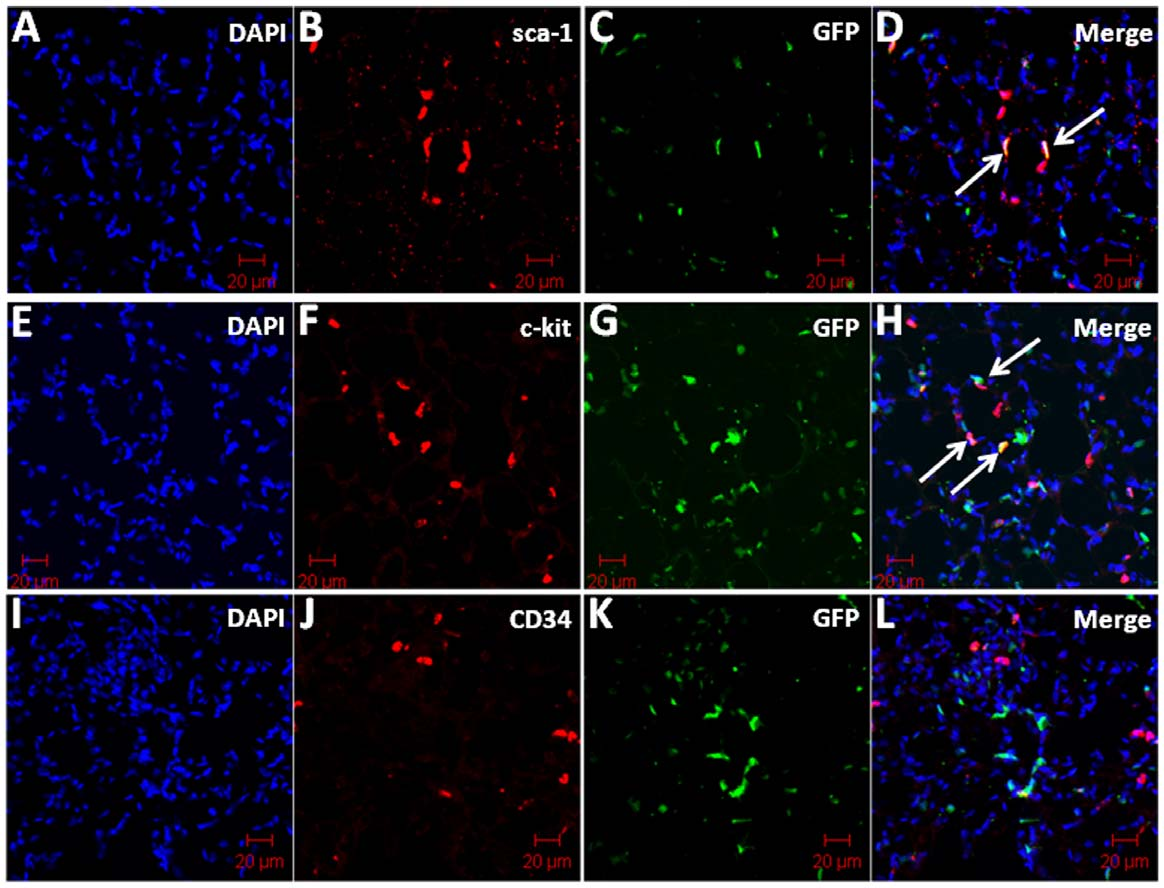
GFP and cellular markers sca-1 and c-kit co-localize in AAV-HIMF-treated bone marrow transplant recipients. Frozen lung sections from bone marrow transplant recipients treated with AAV-HIMF (2.5×10^10^ VP, 14d) were stained with antibodies for (B) sca-1, (F) c-kit, or (J) CD34 (red). (C, G, K) GFP signal was obtained through direct visualization (green). (A, E, I) Cell nuclei were counterstained with DAPI (blue). (D, H, L) The arrows in the merged images demonstrate co-localization of GFP with the cellular markers. Scale bar: 20 µm.

**Figure 6:**
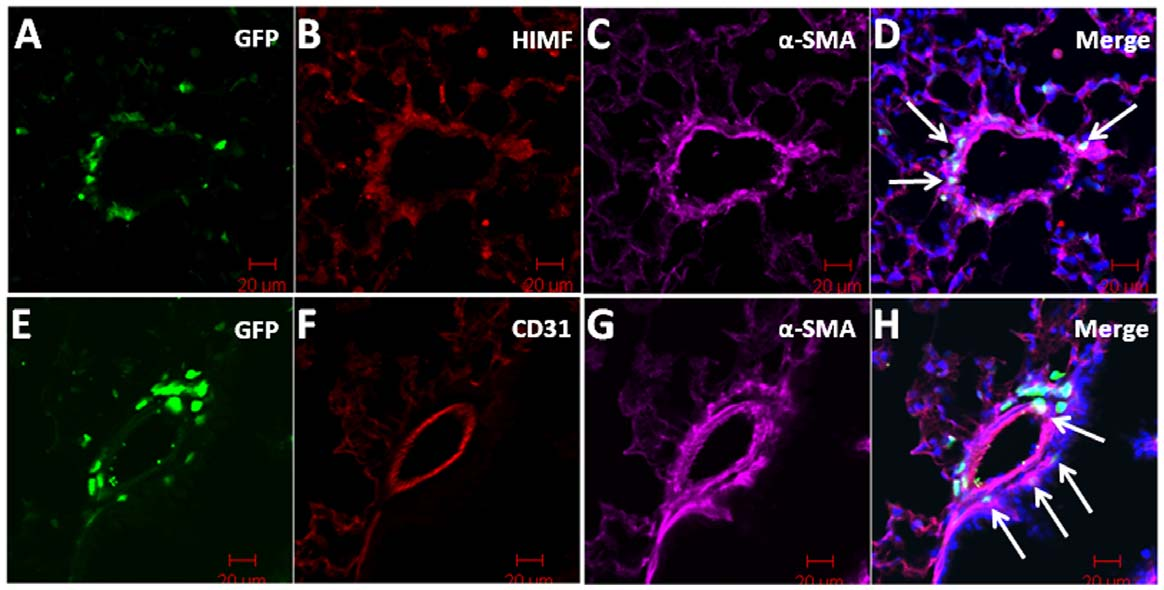
GFP^+^ cells were recruited to the smooth muscle layer of the pulmonary vasculature in AAV-HIMF-treated mice. GFP was detected through direct visualization (A, E; green). HIMF and α-smooth muscle actin (α-SMA) were detected with anti-HIMF and anti-α-SMA primary antibodies and visualized with rhodamine-conjugated anti-rabbit IgG secondary antibodies (B; red) and Cy5-conjugated anti-mouse IgG secondary antibodies (C; pink), respectively. Arrows in the merged image indicate co-localization of GFP, HIMF, and α-SMA (D). Lung sections were stained with anti-CD31 antibodies and visualized with rhodamine-conjugated anti-rat IgG antibodies (F; red) and anti-α-SMA antibodies and visualized with Cy5-conjugated anti-mouse IgG antibodies (G, pink). (F) Arrows in the merged image indicate co-localization of GFP and α-SMA. Cell nuclei were stained with DAPI (D, H; blue) Scale bar: 20 µm.

To determine the phenotype of recruited cells in AAV-HIMF-treated lungs, we used the markers sca-1, c-kit, CD34, CD31, and α-SMA. The markers sca-1 and c-kit indicate that the cells are of hematopoietic lineage and have multipotent potential; CD31 and CD34 are prominent markers for cells of endothelial lineage. Finally, α-SMA indicates cells of mesenchymal origin. As shown in Figure 5, GFP^+^ BMD cells recruited to the vasculature were sca-1^+^ (Figure 5B, D) and c-kit^+^ (Figure 5F, H). Some sca-1^+^ and c-kit^+^ cells contained no GFP signal; these cells are likely unlabeled BMD cells, as not all of the BMD cells from the GFP transgenic donor express GFP [13]. Surprisingly, none of the GFP^+^ cells associated with the vasculature were also CD34^+^ (Figure 5J, L). To determine at which layer of the vasculature the GFP^+^ cells were located, we performed immunohistochemical staining for both α-SMA (vascular smooth muscle) and CD31 (endothelium). As shown in Figure 6, the GFP^+^ cells appeared to be recruited to the smooth muscle layer of the vasculature. GFP and α-SMA signals colocalized in many vascular cells that anatomically appeared to associate with the smooth muscle layer (Figure 6C, D; arrows). These signals also co-localized with HIMF (Figure 6B). There was no apparent co-localization between GFP and the endothelial cell marker, CD31 (Figure 6A, B).

### HIMF-stimulated HMSC chemotaxis is Akt/PI-3K-dependent

**Figure 7:**
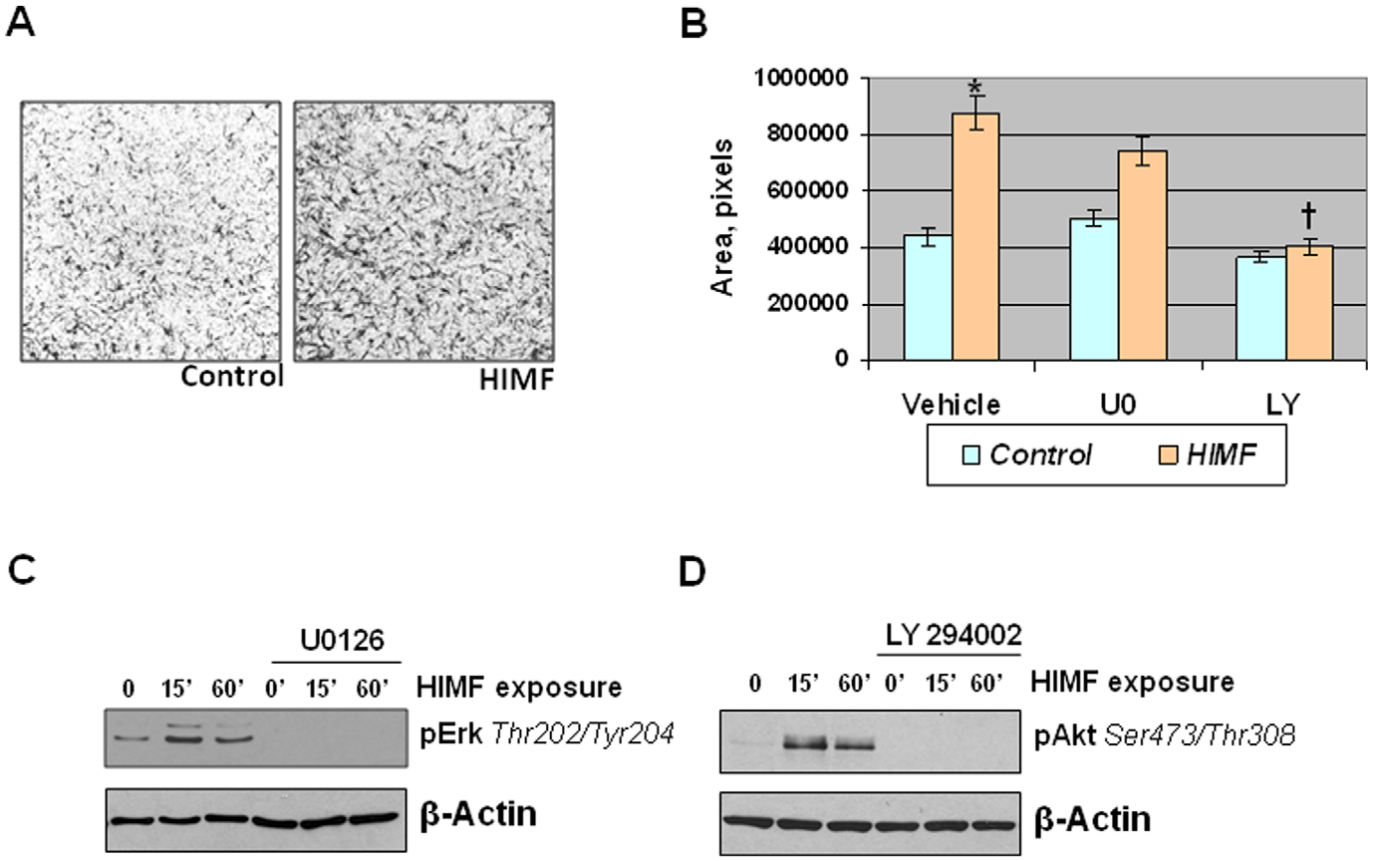
HIMF-stimulated HMSC migration is PI-3K-dependent. A: HMSCs (10^5^ cells) were cultured in the upper chamber of a transwell plate; the lower chamber held medium containing BSA (control) or HIMF (100 nM). After 24 h, the cells were fixed and stained by Coomasie blue solution. B: HMSCs were grown as described in A, but cells were pretreated with vehicle, U0126 (10 µM), or LY294002 (10 µM). Migrated cells were quantified and results were reported as mean (±SEM) of area (pixels). *Significant increase vs. vehicle control at *P*<0.05. ^†^Significant decrease vs. HIMF stimulation alone at *P*<0.05. C, D: HMSCs were cultured to approximately 70% confluence, serum and growth factor starved overnight, and then exposed to HIMF (100 nM) or vehicle for up to 60 min in the presence or absence of ERK1/2 MAPK inhibitor U0126 (10 µM) or PI-3K inhibitor LY294002 (10 µM). Cells were lysed and proteins were resolved with SDS-PAGE and transferred to nitrocellulose membranes. The membranes were probed with rabbit anti-phospho-ERK1/2 (Thr202/Tyr204) (C) or rabbit anti-phospho-Akt (Ser473/Thr308) (D), followed by HRP-conjugated anti-rabbit IgG antibodies, and developed with ECL. To ensure equal loading and transfer, blots were stripped and reprobed with anti-β-actin.

To examine if HIMF has a direct effect on mesenchymal stem cells, we performed a cell migration assay using HMSCs. For these experiments, HMSCs were cultured in transwell plates in the presence of vehicle or recombinant HIMF (100 nM) for 24 h. HIMF increased HMSC migration approximately 2-fold (Figure 7). We have previously shown that HIMF can induce activation of both the Akt/PI-3K pathway and the ERK1/2 MAPK pathway *in vitro* [3]. To determine if HIMF activated these pathways in HMSCs, we treated cultured HMSCs that had been serum and growth factor starved overnight with vehicle or HIMF (100 nM) for 15 or 60 min. The addition of HIMF activated both the PI-3K and ERK1/2 MAPK pathways in a time-dependent manner (Figure 7C, D). Because HIMF induced cell migration and activated these signaling pathways in HMSCs, we wanted to determine if one or both of these pathways were involved in HIMF-induced cell migration. Preincubation of HMSCs with the PI-3K inhibitor LY294002 (10 µM) returned HIMF-induced cell migration to baseline levels (Figure 7B); inhibition of ERK1/2 MAPK with the pharmacological inhibitor U0126 (10 µM) had a slight, but not statistically significant effect (Figure 7B). In Figure 7C and 7D, we demonstrate that both LY294002 and U0126 successfully entered the HMSCs and blocked the appropriate signaling pathway.

## Discussion

In the current study, we examined the possibility that HIMF/FIZZ1/RELMα acts as a chemokine to induce BMD cell recruitment to the remodeling pulmonary vasculature. To address this question, we transplanted bone marrow from GFP^+^ transgenic mice [13] into lethally irradiated wild-type recipients, subjected the recipients to models of PH, and tracked BMD cell migration [14] in the pulmonary vasculature. The results show that both chronic hypoxia and AAV-HIMF introduction induce PH-like vascular remodeling and the engraftment of BMD cells to the pulmonary vasculature. Further analysis of the recruited BMD cells in AAV-HIMF-treated mice revealed that many of these cells expressed c-kit, sca-1, and α-SMA and lacked expression of CD31 and CD34. This profile indicates that the cells are likely mesenchymal in origin and have the capacity to differentiate into myofibroblast-like cells and possibly vascular smooth muscle cells. We also show that HIMF has a direct effect on HMSCs by increasing cell migration in a PI-3K-dependent manner. Together, these findings demonstrate that HIMF/FIZZ1/RELMα plays an important role in the recruitment and engraftment of BMD cells in pulmonary vascular remodeling.

We have previously described HIMF expression in proliferating cell nuclear antigen (PCNA)-positive cells, vascular smooth muscle cells, and endothelial cells of the remodeling vasculature in animals with chronic hypoxia-induced PH [3]. We have also demonstrated that AAV-HIMF pulmonary gene transfer leads to HIMF expression in the vascular smooth muscle and endothelium of lung vessels (all sizes), bronchial epithelial cells, and alveolar type II cells [7]. The HIMF expression pattern in bone marrow transplant recipients used in this study was consistent with our previous work that showed increased HIMF in the actively dividing pulmonary vascular endothelial and smooth muscle cells following hypoxic exposure or pulmonary AAV-HIMF treatment [3], [7]. We also have recently demonstrated that HIMF plays a key role in hypoxia-induced pulmonary vascular remodeling; we can induce pulmonary vascular remodeling and the hemodynamic and cardiac hypertrophic changes of PH by pulmonary gene transfer of HIMF [7]. In the current study, we demonstrate that hypoxia and intranasal instillation of AAV-HIMF induce the same pattern of muscularization of small pulmonary arteries.

It is largely unknown whether the pulmonary vascular remodeling involves the proliferation of resident vascular cells, transition of other resident lung cells to stem cells critical to remodeling, or recruitment of BMD cells and stem cells to the lung. This is an area of considerable controversy. Many recent studies have shown that BMD cells are involved in tissue remodeling and repair of several organs, including the lung [20]. These cells have also been shown to be localized in atherosclerotic lesions of the vasculature [21], [22]. In fact, human patients who receive bone marrow transplants display engraftment of BMD cells within their vasculature [23]. In our study, we demonstrate that GFP^+^ BMD cells are recruited to the pulmonary vasculature following chronic hypoxia or overexpression of HIMF in the lung. In fact, there are nearly twice as many GFP^+^ BMD cells associated with the vessels from mice exposed to chronic hypoxia or AAV-HIMF. In pulmonary vessels from normoxia/AAV-null control mice, there is almost never more than one GFP^+^ cell associated with an individual vessel. These GFP^+^ cells are rarely incorporated into the media layer in the vessels of these mice. In the experimental groups (chronic hypoxia or AAV-HIMF), it is common for some pulmonary vessels to have multiple associated GFP^+^ cells with these cells frequently localizing to the medial layer of the vessel. In several cases, these BMD cells aggregate around previously endothelial-only capillary vessels; neomuscularization of these vessels are a key component of the pathogenesis of PH.

Recent evidence has suggested a potential role for the recruitment of BMD progenitor cells to the remodeled pulmonary vasculature associated with PH [11], [12], [24]. An initial study by Davie *et al.* [11] revealed an increased number of c-kit-expressing cells in the circulation and in the pulmonary vasculature of hypoxic calves. C-kit, also known as CD117, is the receptor for a cytokine called stem cell factor; it is expressed on the surface of BMD cells with multipotent potential [25]. A subsequent study by Hayashida *et al.* [24] demonstrated that mice exposed to chronic hypoxia for 4 or 8 weeks displayed increased infiltration of BMD cells in the lung and lung vasculature compared with normoxic control. Many of these recruited BMD cells were α-SMA^+^. Hypoxia does not seem to be the only stimulus for this process; in the monocrotaline inflammatory model of PH, many BMD cells were recruited to the pulmonary vasculature, some of which were α-SMA^+^ [12]. A detailed examination of AAV-HIMF-treated lungs showed that many of the recruited cells stained positive for the cellular markers c-kit and sca-1. This finding would indicate that these BMD cells have multipotent potential, including the ability to differentiate into mesenchymal-like cells; these differentiated cells could then participate in the observed pulmonary vascular remodeling. The fact that these cells were negative for the endothelial progenitor markers CD31 and CD34 strengthens the possibility that these cells will likely differentiate into mesenchymal-like cells. Several of the GFP^+^ cells associated with the pulmonary blood vessels also expressed α-SMA, indicating a mesenchymal lineage and suggesting the possible transition to myofibroblasts and vascular smooth muscle cells. The fact that many of the smallest neomuscularized vessels expressed only GFP^+^ cells in their new medial layer, strongly suggests a functional role for these cells. This work supports the ability of HIMF to recruit BMD cells to the vascular wall in the remodeling associated with the development of PH.

We have recently demonstrated that HIMF is chemotactic for murine BMD cells in culture [5] and that the mechanism involves HIMF binding to BTK, resulting in BTK autophosphorylation and intracellular movement of BTK to the migrating cell process. Here, we showed that HIMF expression in the lung can recruit BMD cells to the remodeling pulmonary vasculature and that HIMF induces chemotaxis of HMSCs in culture. These results are consistent with our earlier findings and those of previously published reports [11], [24] in which chronic hypoxia induced vascular remodeling. Intranasal AAV-HIMF treatment elicited similar results.

Our finding that HIMF was chemotactic for HMSCs in this study raises the possibility that HIMF directly recruits mesenchymal stem cells to the remodeling pulmonary vasculature. Previous studies have demonstrated the existence of circulating BMD smooth muscle cells and the potential vascular engraftment of these cells in human disease [21], [23]. In our system, we showed that these cells are of mesenchymal lineage and directly engraft into the vascular wall. It is important to note the possibility that HIMF is driving these recruited cells to the mesenchymal lineage. HIMF has been shown to play a key role in the transition of fibroblasts to myofibroblasts in experimentally-induced pulmonary fibrosis [8]. It is likely that the cells that are currently c-kit^+^ and sca-I^+^ will transition into vascular smooth muscle cells in the remodeling vasculature.

A receptor for HIMF or its related molecules has not yet been described, although a few signaling pathways have been identified. We have previously reported that HIMF activates the PI-3K pathway in pulmonary vascular smooth muscle cells in a dose- and time-dependent manner [3]. We have also demonstrated that HIMF activates the Akt/PI-3K pathway in endothelial cells; this activation plays a critical role in HIMF-induced endothelial migration and tubule formation [6]. Other groups also have shown this pathway to be activated in primary lung fibroblasts as well as endothelial and lung epithelial cell lines [9], [10]. Here we showed that HIMF activates the PI-3K pathway in a time-dependent manner in primary HMSCs and that this pathway is involved in HIMF stimulated cell migration. One signaling pathway that activates the Akt/PI-3K pathway in several cellular systems is BTK [26]. Our previous studies have shown that HIMF is a binding partner for BTK as well as an activating agent in murine BMD cells [5]; intracellular movement of BTK to the migrating cell process is essential to HIMF-induced cell migration of these cells. It is possible that HIMF is activating Akt/PI-3K through the BTK pathway in this system. HIMF also activated ERK1/2 MAPK in a time-dependent manner in HMSCs, but this pathway did not appear to be involved in the HIMF-stimulated cell migration process.

In summary, the current study demonstrates that pulmonary gene transfer of HIMF induces pulmonary vascular remodeling and the recruitment of BMD cells to the pulmonary vasculature similar to that of chronic hypoxia. Cells that were recruited to the vasculature were c-kit^+^, sca-1^+^, and α-SMA^+^, but CD31^−^ and CD34^−^; these results suggest that these recruited cells are BMD and mesenchymal in origin or have the potential to differentiate into mesenchymal-like cells that participate in pulmonary vascular remodeling. The study also shows that HIMF has direct action on HMSCs by inducing PI-3K-dependent chemotaxis. Taken together, these data suggest that HIMF plays an important role in the recruitment of BMD cells to the remodeling pulmonary vasculature.
